# Trichloromethane fraction of *Incarvillea compacta* induces lytic cytotoxicity and apoptosis in Epstein-Barr virus-positive gastric cancer AGS cells

**DOI:** 10.1186/s12906-016-1331-6

**Published:** 2016-09-05

**Authors:** Lijing Zhang, Haifeng Wu, Guibo Sun, Xudong Xu, Xiaobo Sun, Li Cao

**Affiliations:** Institute of Medicinal Plant Development, Chinese Academy of Medical Sciences & Peking Union Medical College, 151 Malianwa North Road, Haidian District Beijing, 100193 China

**Keywords:** Gastric cancer, *Incarvillea compacta*, EBV, Cell cycle, Apoptosis

## Abstract

**Background:**

*Incarvillea compacta* Maxim. has been used to treat stomach disease in Tibet for many years. The objectives of this study were to explore the anti-cancer ability of trichloromethane fraction of *I. compacta* Maxim. roots (IC-TCL, R2) in EBV positive AGS cancer cells and its effects on cell cycle arrest, apoptosis and lytic induction.

**Methods:**

MTT and trypan blue assays were to detect the inhibitory effects of different fraction in different cell lines. Hoechst 33342 staining, Annexin V-PE/7-AAD staining and DIOC_6_ staining were used to detect the apoptosis induction effects of R2. Western blot experiments were used to detect the expression of apoptosis related proteins BAX and Bcl-2, EBV lytic related proteins BZLF1 and BMRF1, cell cycle regulation related proteins Cyclin D1 and RB after R2 treatment. Cell cycle arrest was analyzed by flow cytometry.

**Results:**

MTT and trypan blue assays revealed that R2 could significantly reduce cell viability in a dose-dependent manner in EBV positive AGS cells compared with non-EBV infected AGS and other cancer cell lines, whereas n-BuOH and H_2_O fractions showed non-inhibitory effects in tested cancer cells. R2 could decrease mitochondrial membrane potential and the expression of Bcl-2, while increase the expression of BAX. R2 could also induce EBV lytic replication by activating mRNA levels of BZLF1, BRLF1 and BMRF1. Protein expressions of BZLF1 and BMRF1 were also increased after R2 treatment. Cell cycle analysis showed that R2 treatment could induce G0/G1 phase arrest. The expression of Cyclin D1 decreased, while Rb increased.

**Conclusions:**

These results demonstrated that R2 could inhibit the proliferation of AGS-EBV cancer cells by inducing EBV lytic replication, apoptosis and G0/G1 arrest, through the regulation of related proteins. Therefore, R2 could be used as a potential treatment in AGS-EBV cells.

**Electronic supplementary material:**

The online version of this article (doi:10.1186/s12906-016-1331-6) contains supplementary material, which is available to authorized users.

## Background

Gastric cancer was the third leading cause of global cancer deaths, causing a major health issue [1]. In recent years, with the development of tumor molecular biology techniques and understanding of pathogenesis, the molecular targeted therapies have been developed according to the reveal of oncogenic pathways [2–4]. Hence, there is a need to increase the efficacy of treatment and to reduce the side effects in gastric cancer therapy. Approximately ten percent of gastric cancer was infected with Epstein-Barr virus (EBV) [5]. EBV is a member of the γ‑herpesvirus family which infects 90 % of the world population [6]. The EBV life cycle includes latent and lytic form [7]. EBV persists in EBV positive malignancies as latent form; when the lytic cycle of EBV was activated, larger numbers of lytic proteins are expressed at high levels [27]. Lytic EBV replication damages the host cancer cells, which provides a potential therapeutic target for EBV associated cancers [28, 29]. The switch from latent to lytic EBV infection is mediated by expression of the two EBV immediate-early viral proteins, BZLF1 and BRLF1, which could activate the complete cascade of lytic viral gene expression [8, 9]. These proteins activate the viral early genes, resulting in a cascade of events that lead to the activation of lytic replication. For the lytic form of EBV infection, the host immune responses were triggered against EBV and the host cancer cell may be killed [10]. EBV-associated malignancies generally have only a very low level of lytic EBV gene expression without chemotherapy [11]. Several therapeutic strategies could induce the lytic form of EBV infection by activating EBV lytic genes for tumor cells killing have been reported [12, 13]. For example,Romidepsin showed the ability to activate the lytic cycle of EBV and lead to cell death in EBV positive gastric cancer cells [14]. Taking together, these findings suggested that EBV itself could serve as a target for the killing of tumor cells.

Plants with various curative properties have received attentions in the area of pharmacology [15] Medicinal plants have played important roles in anti-cancer drug discovery [24]. Natural products have been involved in the development of approximately 75 % of anticancer agents from 1981 to 2010 [16]. Therefore, exploring the possible mechanism of bioactivities of traditional used plants could promote the development of pharmaceutical products. *Incarvillea* belongs to the family Bignoniaceae, genus Incarvillea. *Incarvillea compacta* Maxim. is a perennial herb mainly distributed in Tibet, which has been traditionally used for treating dyspepsia and gastralgia for centuries [17]. So far, there have been studies on the chemical composition of other species of genus Incarvillea [18–21], which show antioxidant activities and life span prolonging, inhibitory effects on multiple kinase targets and their downstream pathways activated by solar UV in vitro and in vivo [25, 26]. However, no pharmacological studies in stomach disorder treatment are available so far. Besides, the potential value of the herb in treating gastric cancer should not be ignored. Our previous phytochemical investigations on the species disclosed the presence of phenylethanoid glycosides in n-butyl alcohol fraction exhibiting hepatoprotective activity [22]. Thus, the present study was initiated to investigate anticancer effects of *I. compacta.*

In this study, we analyzed the cytotoxic effects of various fractions of *I. compacta* in stomach (AGS, AGS-EBV, BGC-823), EBV-transformed B-cell lines (lymphoblastoid cell lines, LCL), liver (HepG-2), leukemia (K562), cervix (HeLa), lung (A549) and prostate (PC3 and DU145) cancer cells. The most effective fraction (trichloromethane fraction, IC-TCL, R2) in AGS-EBV cells growth inhibition was further evaluated for the induction of apoptosis, EBV lytic, and cell cycle arrest. We confirmed that R2 induce the expressions of EBV lytic genes in AGS-EBV cells and EBV-transformed B-cell lines (LCL), resulting in EBV-positive cells death in vitro. These findings indicated that R2 may be used as a novel agent in treating EBV-positive tumors.

## Methods

### Plant materials

*I. compacta* roots were collected in Huzhu County, Qinghai Province, China in July 2013, and identified by Prof. Xiao-Feng Zhang of the Department of Tibetan medicines, Northwest Institute of Plateau Biology, Chinese Academy of Sciences. A voucher specimen (NO. 130718) was deposited at the Key Laboratory of Bioactive Substances and Resource Utilization of Chinese Herbal Medicine, Ministry of Education, Institute of Medicinal Plant Development, Peking Union Medical College and Chinese Academy of Medical Sciences.

### Preparation of plant extract and fraction

Dried and coarsely powered plant roots material of *I. compacta* (1.1 kg) was extracted three times with 90 % ethanol (3 × 3 L) at room temperature. Removal of the ethanol under reduced pressure yielded the *I. compacta* ethanolic extract (IC-ET). The practical yield of IC-ET was 8.90 %. The IC-ET (90 g) was suspended in distilled water (1 L) and then the suspension was partitioned with trichloromethane and n-BuOH, successively, yielding the trichloromethane fraction (IC-TCL), the n-BuOH fraction (IC-BT), and the H2O fraction (IC-R). Each fraction was concentrated using rotary evaporator in vacuum, and completely dried. The yield of IC-TCL, IC-BT, and IC-R was 24.4 %, 36.7 %, and 33.3 %, respectively. For biological assays, IC-TCL, IC-BT, and IC-R were dissolved in pure dimethyl sulfoxide and subjected to serial dilution so that the final concentration of DMSO in solution was less than 1 %.

### Instrumentations and analytical conditions

#### Ultra-high performance liquid chromatography (U-HPLC)

Chromatography was performed on a Dionex UltiMate 3000 U-HPLC system consisted of an auto-sampler, a quaternary pump, and a column oven (Thermo, Markham, Ontario, Canada). The chromatographic separation was performed on a Waters Acquity BEH C18 column (2.1 mm × 100 mm, 1.7 μm, Waters Corporation, Milford, MA). The mobile phase was comprised of 5 mM ammonium formate in water (solvent A) and 5 mM ammonium formate in methanol (solvent B) at a flow rate of 0.3 mL/min. The gradient elution program was as follows: 5 % B – 25 % B at 0–2 min; 25 % B – 100 % B at 2–30 min; 100 % B – 100 % B at 30–35 min. The column oven temperature and the auto-sampler temperature were maintained at 30 °C and 4 °C, respectively. The sample injection volume was 5 μL.

### Mass spectrometer

QExactive Orbitrap FTMS mass spectrometer equipped with an electrospray ionization (ESI) source (Thermo, Markham, Ontario, Canada) was connected to the UHPLC system via an electro spray ionization source (ESI) interface. The ESI source was operated in a positive ionization mode at a capillary voltage of 3.5 kV. Nitrogen was used as the desolvation gas (600 L/h) and cone gas (50 L/h). The temperatures of the source and desolvation were set at 150 and 400 °C, respectively. Nitrogen was used as the collision gas, and the collision energy was 14 eV.

### Reagents and antibodies

Dulbecco’s Modified Eagle’s Medium (DMEM), Ham’s F12 medium, trypsin, penicillin, streptomycin, fetal bovine serum (FBS) were purchased from Gibco (CA, USA), and 3-(4,5-dimethylthiazol-2-yl)-2, 5-diphenyltetrazolium bromide (MTT), DMSO, Hoechst 33342, DIOC_6_, RNase A, propidium iodide (PI), Trizol and trypan blue were purchased from Sigma-Aldrich (MO, USA). Dihydroethidium was bought from Beyotime Biotech (Jiangsu, China). The Annexin V-PE/7-AAD apoptosis detection kit was obtained from KeyGEN Biotech (Jiangsu, China). Antibodies against Bax, Bcl-2, Cyclin D1, BZLF1 and BMRF1 were obtained from Santa Cruz Biotechnology (CA, USA). Bax ((6A7) sc-23959) is a mouse monoclonal antibody raised against the N-terminal residues 12–24 common to human, mouse and rat Bax protein. Bcl-2 ((C-2) sc-7382) is a mouse monoclonal antibody raised against amino acids 1–205 of Bcl-2 of human origin. Cyclin D1 ((H-295) sc-753) is a rabbit polyclonal antibody raised against amino acids 1–295 representing full length cyclin D1 of human origin. EBV Ea-D ((1108-1) BMRF1, sc-69679) is a mouse monoclonal antibody raised against affinity purified early antigen polypeptides from induced Raji cells precipitated with African Burkitt’s lymphoma sera. EBV ZEBRA ((BZ1) BZLF1, sc-53904) is amousemonoclonal antibodyraised against full-length recombinant EBV ZEBRA protein. Antibodies against Rb, and β-actin were purchased from Cell Signaling Technology (MA, USA). Rb (4H1) Mouse mAb #9309 is produced by immunizing animals with a Rb-C fusion protein containing residues 701–928 of human Rb. β-actin (8H10D10) Mouse mAb #3700 is produced by immunizing animals with a synthetic peptide corresponding to amino-terminal residues of human β-actin The cECL Western Blot Kit and SYBR Green Premix detection system were obtained from CoWin Biotech (Beijing, China). All the chemical reagents used were of the highest grade.

### Cell culture

AGS (human gastric carcinoma cells), wild-type EBV positive AGS cell line (AGS-EBV) and wild-type EBV-transformed B-cell lines (lymphoblastoid cell lines, LCL) were obtained from Professor Wenhai Feng (College of Biological Sciences, China Agricultural University, China). GES-1 cell line was purchased from Cancer Institute & Hospital, Chinese Academy of Medical Sciences (China). Other cell lines used were from Chinese Academy of Sciences (China). Cells were cultured in Ham’s F-12 medium (AGS and AGS-EBV cell lines), RPMI 1640 medium (LCL, K562) and DMEM medium (other cell lines) containing 10 % fetal bovine serum, 100 U/ml penicillin and 100 μg/ml streptomycin at 37 °C with 5 % CO_2_. Medium contain DMSO were used as vehicle control.

### Cell viability and cytotoxicity assay

MTT assay was used to determine the cell viabilities after the treatment of tested fractions. AGS, AGS-EBV, HeLa, BGC-823, GES-1 cells (6 × 10^3^ cells/well); WPMY, SV-HUC-1, DU145, PC3 cells (7 × 10^3^ cells/well); A549, HepG-2 cells (8 × 10^3^ cells/well); LCL, K562 cells (9 × 10^3^ cells/well) were seeded in triplicate in 96-well plates and cultured at 37 °C for 24 h. Cells were treated by different fractions in various concentrations (DMSO, 2.5, 5, 10, 20, 40 μg/mL). After 24 h treatments, MTT (5 mg/mL) was added to each well for another 4 h. The medium were then removed and 150 μL DMSO was added. The absorbance was measured at 570 nm using the Microplate Reader (Bio Tek, America). Cell viability was expressed as the ratio of surviving cells in each group to control group.

Trypan blue exclusion was used to examine the numbers of dead cells in each group. AGS cells and AGS-EBV cells (1 × 10^6^ cells/well) were plated in 6-well plates for 24 h and then treated with various concentrations of R2 (DMSO, 2.5, 5, 10, 20 and 40 μg/mL) for 24 h, 48 h and 72 h. After harvesting, the cells were suspended in PBS and mixed with 0.4 % trypan blue solution. The number of viable cells and dead cells were counted under the light microscope.

### Detection of apoptotic cells

Apoptotic cells were detected by Hoechst 33342 staining and Annexin V-PE/7-AAD detection. AGS-EBV cells were cultured in 96-well plates and treated with R2 (DMSO, 5, 10, and 20 μg/mL) for 24 h. After washed with PBS, cells were stained with Hoechst 33342 (10 μg/mL) for 10 min. Morphology changes in nuclear were observed using Image Xpress Micro imaging system (Molecular Devices, USA).

AGS-EBV cells (1 × 10^6^ cells/well) were seeded in 6-well plates for 24 h and then treated with various concentrations of R2 (DMSO, 5, 10 and 20 μg/mL) for 24 h. After washing twice with PBS, the cells were stained by an Annexin V-PE/7-AAD apoptosis kit (KeyGEN Biotech, Nanjing, China) according to the manufacturer’s instructions. Stained cells were detected and analyzed using flow cytometry (Becton Dickinson, USA).

### Detection of mitochondrial membrane potential

Changes in the mitochondrial membrane potential after R2 treatment were measured by flow cytometry using DIOC_6_. Cells were treated with R2 (DMSO, 5, 10, and 20 μg/mL) for 24 h. Cells were then harvested and incubated with DIOC_6_ (5 μM) for 30 min in the dark at 37 °C. After washing with PBS, cells were analyzed by flow cytometry.

### Cell cycle analysis

Cell cycle phase distributions were measured by staining DNA with Propidium Iodide. AGS-EBV cells were seeded in 6-well plates and treated with R2 (DMSO, 5, 10, and 20 μg/mL) for 24, 48 and 72 h. Then harvested the cell and fixed in 70 % ethanol overnight at -20 °C. After washing 3 times with PBS, incubated the cells with RNase A (Amresco, USA) for 20 min. Then the cells were stained with 50 μg/mL PI for 10 min in the dark at room temperature. DNA contents were detected by flow cytometry and analyzed by ModFit LT 4.0.

### Western blot

AGS-EBV and LCL cells were exposed to R2 (DMSO, 5, 10, and 20 μg/mL) for 24 h. After collection, cells were lysed in lysis buffer and protein concentrations were determined by BCA method. Protein samples (40 μg) were separated by SDS–PAGE gel electrophoresis and electrically transferred onto PVDF membranes. After blocking with 5 % non-fat milk solution for 1 h, the membranes were incubated with primary antibody at 4 °C overnight. Later, washed away the primary antibody with TBST and incubated with the HRP-conjugated secondary antibody at room temperature for 1 h. The protein bands were visualized by cECL. The level of β-actin for each sample was used as a control.

### RT-PCR detected the expression of specific mRNA related with EBV lytic replication

The changes of mRNA expression after R2 treatment in AGS-EBV cells were quantified by real-time polymerase chain reaction (RT-PCR). AGS-EBV cells were exposed to R2 (DMSO, 5, 10, and 20 μg/mL) for 24 h. Trizol was used to lysis cells. After RNAs were extracted, we used Nanodrop 2000 (Thermo scientific) to quantity RNA. The sequences of primers were AAATTTAAGAGATCCTCGTGTAAAACATC (sense) and CGCCTCCTGTTGAAGCAGAT (anti-sense) for BZLF1; ATGGAACATGCGTCGTTG (sense) and AATGGCCACGCTCAACAT (anti-sense) for BRLF1; CAACACCGCACTGGAGAG (sense) GCCTGCTTCACTTTCTTGG (anti-sense) for BMRF1, TTGCCATCAATGACCCCTTCA (sense) and CGCCCCACTTGATTTTGGA (anti-sense) for β-Actin. β-Actin was served as an internal reference. The mRNA expression levels were measured by SYBR Green Premix detection system (Cwbio, China). The PCR conditions were 95 °C 20 s;95 °C 3 s、60 °C 30 s (40 cycles).

### Statistical analysis

All data were analyzed by IBM SPSS statistics 19. The differences in the means between groups were compared by t-tests. The statistically significant between groups was defined as **p < 0.01 or *P < 0.05. Results were expressed as mean ± SD.

## Results

### The effects of growth suppression and death induction of trichloromethane fraction of *I. compacta* Maxim. roots (IC-TCL, R2) in EBV positive human gastric cancer cells (AGS-EBV)

**Fig. 1:**
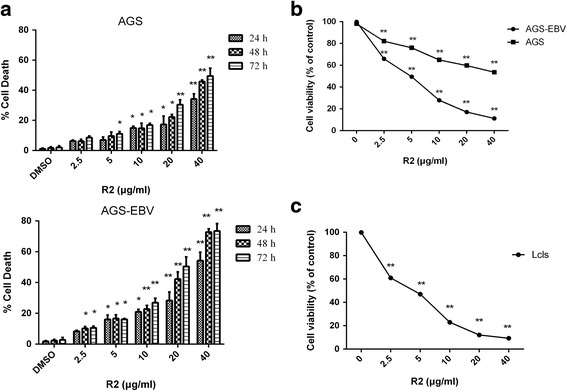
Inhibition effects of R2 on cell viabilities. Effects of R2 on the viabilities of EBV positive human AGS gastric cancer cells and AGS by trypan blue assay (**a**) and MTT assay (**b**). Effects of R2 on the viabilities of EBV-transformed B-cell lines (LCL) (**c**). Cells were exposed to R2 (0, 2.5, 5, 10, 20, 40 μg/mL) for 24 h, 48 h or 72 h for trypan blue assay and 24 h for MTT assay. R2 suppressed cell viability and induced AGS-EBV and LCL cell death, while being less cytotoxic to AGS cells. The data showed the mean value of three independent tests and were expressed as means ± SD. *P < 0.05 and **P < 0.01 were considered statistically significant

UHPLC-MS chromatogram result of dichloromethane fraction of *I. compacta.* was showed in Additional file 1: Figure S1. First we used MTT assay to explore the anti-proliferative effects of fractions from *I. compacta* roots against commonly observed cancer cell lines: stomach (AGS, EBV-AGS, BGC-823), liver (HepG-2), leukemia (K562), cervix (HeLa), lung (A549) and prostate (PC3 and DU145) cancer. The cell viabilities and fraction concentrations resulting in 50 % growth inhibition (IC_50_) were showed in Fig. 1b, Table 1 and Table 2 of each fraction in all cell lines. The IC_50_ values varied in each cell line for different fractions. R2 showed dose-dependent cytotoxic effects in AGS-EBV cancer cells with IC_50_ of 5.74 μg/mL and 52.24 μg/mL in AGS (Fig. 1b; Table 1). While showed less cytotoxic effects in human normal gastric epithelial cell line GES-1, human normal prostate stromal immortalized cell line WPMY, human normal bladder cell SV-HUC-1 and other cancer cell lines (Table 3). However, other fractions of the roots of the plant showed less cytotoxic effects in all cancer cell lines tested in this research (Table 1 and 2). It indicated that trichloromethane fraction of *I. compacta* roots (R2) was most effective in inhibiting the proliferation of EBV positive AGS cancer cells.

**Table 1:** IC_50_ values of different cancer cell lines after treatment with R2

Compounds	IC50(μM)^a^				
	AGS	AGS-EBV	LCL	HepG2	PC3
IC-TCL	52.24 ± 3.01	5.64 ± 1.14	5.12 ± 1.66	39.73 ± 2.92	81.57 ± 3.29
IC-BT	94.74 ± 3.53	29.57 ± 2.33	42.37 ± 5.31	64.23 ± 2.76	>200
IC-R	163.62 ± 3.85	68.73 ± 2.12	>200	79.58 ± 4.32	96.18 ± 4.67

^a^IC_50_ is the concentration of compound causing 50 % growth inhibition in each cell line. The results showed the mean values of three independent tests

**Table 2:** IC_50_ values of different cancer cell lines after treatment with R2

Compounds	IC50(μM)^a^				
	HeLa	K562	A549	DU145	BGC-823
IC-TCL	54.26 ± 3.18	27.47 ± 2.06	19.96 ± 3.28	50.51 ± 3.98	90.24 ± 2.57
IC-BT	43.27 ± 2.23	38.53 ± 1.97	>200	82.73 ± 1.62	34.78 ± 2.61
IC-R	85.96 ± 3.21	>200	76.53 ± 2.26	37.51 ± 1.77	54.13 ± 2.74

^a^IC_50_ is the concentration of compound causing 50 % growth inhibition in each cell line. The results showed the mean values of three independent tests

**Table 3:** IC_50_ values of different normal cell lines after treatment with R2

Compounds	IC50(μM)^a^		
	GES-1	WPMY	SV-HUC-1
IC-TCL	83.57 ± 2.46	>200	97.65 ± 5.21

^a^IC_50_ is the concentration of compound causing 50 % growth inhibition in each cell line. The results showed the mean values of three independent tests

Then we used trypan blue exclusion to evaluate the cytotoxicity of R2 in AGS and AGS-EBV cells. AGS and AGS-EBV cells were exposure to various concentrations of R2 (0, 2.5, 5, 10, 20, 40 μg/mL) for 24 h, 48 h and 72 h. As shown in Fig. 1a, treatment with 10 μg/mL of R2 for 48 h and 72 h resulted in significant effects on cell death in AGS-EBV, while the number of dead cells slightly changed after R2 treatment in AGS cells. Together, these data suggested that R2 can inhibit the growth and induce the death in AGS-EBV cells while less cytotoxic to AGS cells.

Therefore we choose R2 for further investigation to analyze the anti-cancer mechanisms in EBV positive AGS cancer cells.

### The effect of growth suppression of R2 in LCL cells

Since R2 significantly inhibited the growth of AGS-EBV cells, we then investigated the effect of R2 on the viability of EBV-transformed B-cell lines (LCL). LCL cells were treated with different concentrations of R2, and the cell viability was measured by MTT assay. The results showed that the cell viability of LCL decrease in a dose-dependent manner. Cell viability reduced by 40 % at 2.5 μg/mL and further reduced by 80–90 % at 20 μg/mL R2 treatment (Fig. 1c).

### Apoptosis evaluation using Hoechst staining and Annexin V-PE/7-AAD assay after R2 treatment in AGS-EBV cells

**Fig. 2:**
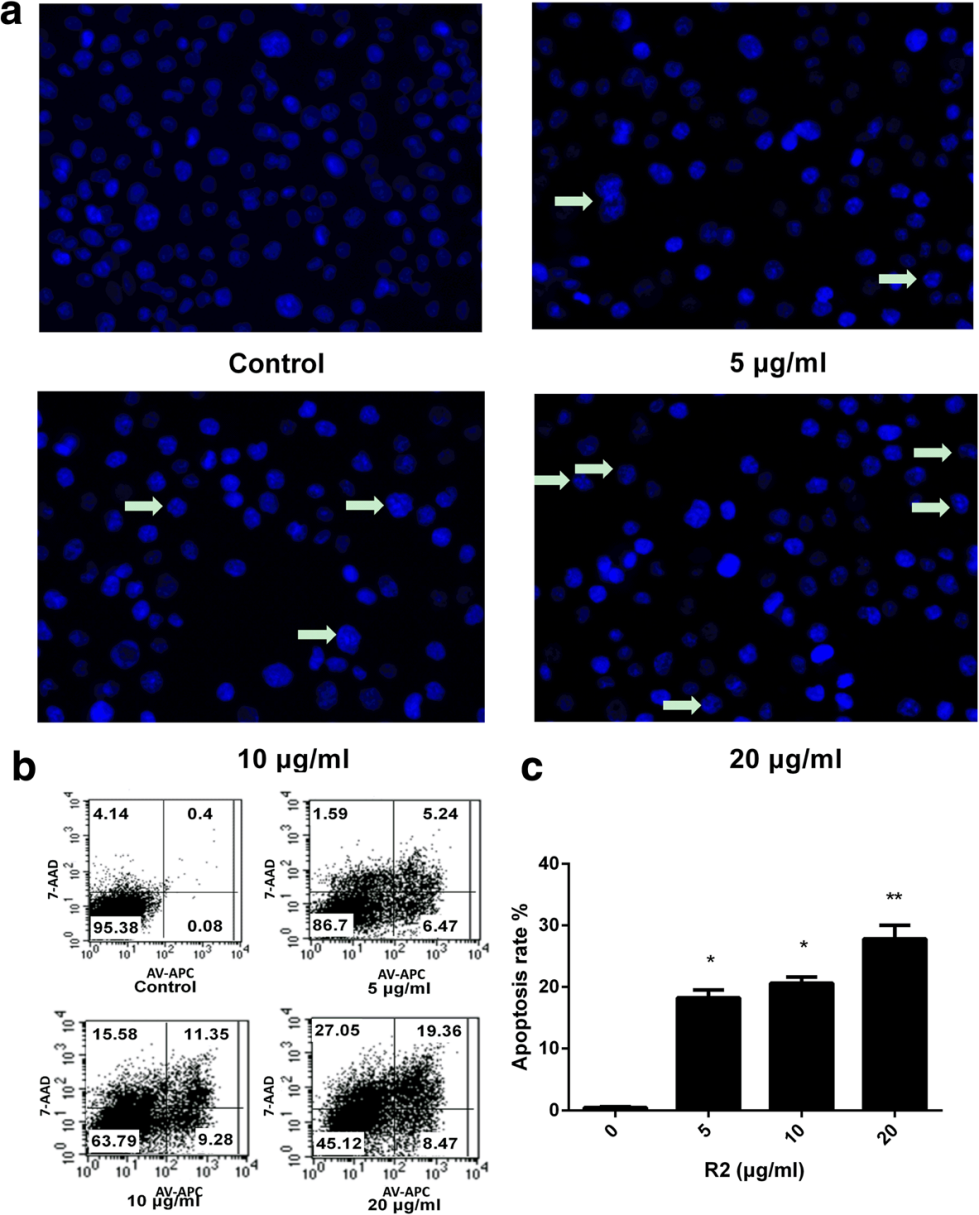
Induction of apoptosis after R2 treatment in AGS-EBV cells. Micrographs show apoptotic cells after treatment with R2 at different concentrations and staining by Hoechst 33342 for 24 h (**a**). R2 induced apoptosis in AGS-EBV cells was also detected by the Annexin V-FITC/7-AAD staining test (**b** and **c**). Cells were treated with R2 (0, 5, 10, 20 μg/mL) for 24 h. DMSO treatment was used as a vehicle control. The apoptotic rates were determined by Annexin V-FITC/7-AAD staining. *P < 0.05 and **P < 0.01 were considered statistically significance

To elucidate whether the cytotoxic effect of R2 was associated with apoptosis induction, we used Hoechst 33342 and Annexin V-PE/7-AAD staining to explore the morphological changes and the numbers of apoptotic cells after R2 treatment. Based on IC_50_ value, we used 5 μg/mL, 10 μg/mL and 20 μg/mL of R2 as treatment concentrations in AGS-EBV cells. In Hoechst 33342 staining, typical morphological change of apoptosis as condensed chromatin was observed when cells were exposure to R2 (Fig. 2a). In Annexin V-PE/7-AAD assay, the population of apoptotic cells (AV+/7-AAD- plus AV+/7-AAD+) increased in a dose-dependent manner (Fig. 2b and c) when compared with vehicle group.

These observations indicated that the proliferation inhibition effect of R2 in AGS-EBV cells may be related with apoptosis induction.

### Effect of R2 on mitochondrial membrane potential in AGS-EBV cells

**Fig. 3:**
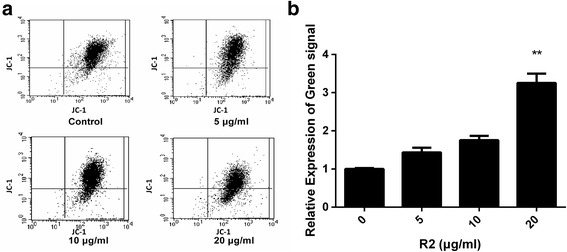
R2 induced mitochondrial membrane potential depolarization (**a** and **b**) in AGS-EBV cells. AGS-EBV cells were cultured in R2 (0, 5, 10, 20 μg/mL) for 24 h. DMSO treatment was used as vehicle control. Cells were labeled with DIOC6 and then analyzed by flow cytometry to detect the levels of mitochondrial membrane potential. Relative expression levels were showed in **b**. Results obtained from a representative experiment are shown (*n* = 3). ** *p* < 0.01 was considered statistically significant

Apoptosis is also marked by the decrease of mitochondrial membrane potential. DIOC_6_ is often used as an indicator to detect Δψ during apoptosis. To further confirm the induction of apoptosis, we then analyzed the changes of mitochondrial membrane potential in AGS-EBV cells. As showed in Fig. 3a and b, R2 treatments induced significant increase of green fluorescence from 9.5 % to 30.93 %, indicating the loss of Δψ. These results further showed that the anti-proliferation effect of R2 is associated with the induction of apoptosis. DIOC_6_ fluorescence was mostly appeared in red indicated that the majority cells were alive in control group.

### Effects of R2 on the expression of apoptosis and cell cycle regulation-related proteins in AGS-EBV cells

**Fig. 4:**
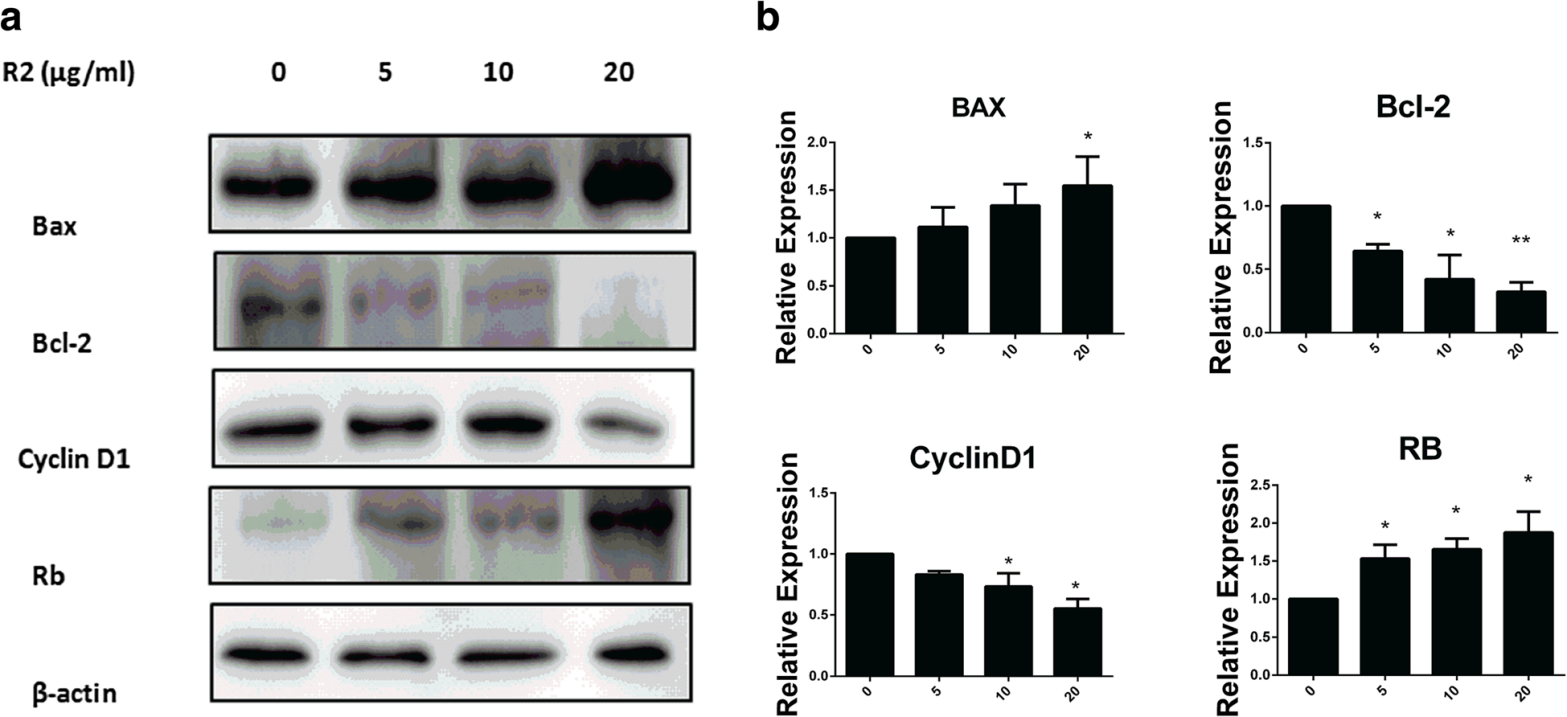
Effects of R2 on the expression of apoptosis and cell cycle related proteins in AGS-EBV cells were detected by western blot. AGS-EBV cells were treated with R2 (0, 5, 10, 20 μg/mL) for 24 h. R2 decreased the expressions of Bcl-2 and cyclin D1, while increased the expressions of Bax and Rb (**a**). Relative expression levels of proteins were showed in (**b**). β-actin was used to confirm equal protein loading. Results obtained from a representative experiment were shown (*n* = 3). *P < 0.05 and ** *p* < 0.01 were considered statistically significant

The Bcl-2 family proteins are critical regulators of apoptosis at the mitochondria and endoplasmic reticulum [23]. Since the results showed that R2 induce apoptosis in AGS-EBV cells, we then analyzed the expression of Bax and Bcl-2 proteins after treated with R2. Western blot results showed Bax protein increased while Bcl-2 protein decreased after treatment (Fig. 4). These results indicated that the apoptosis activated by R2 was related with Bcl-2 family proteins.

To gain further understanding about the molecular mechanisms of cell cycle arrest in AGS-EBV cells, we analyzed the effects of R2 on the expression of G0/G1 phase regulatory proteins. Figure 4 showed that after treatment with R2, the expression of cyclin D1 decreased, while the expression of Rb increased. These results suggested that the changes of protein expression may play important roles in G0/G1 arrest of cell cycle in AGS-EBV cells.

### Effects of R2 on EBV lytic mRNAs expression in AGS-EBV cells

**Fig. 5:**
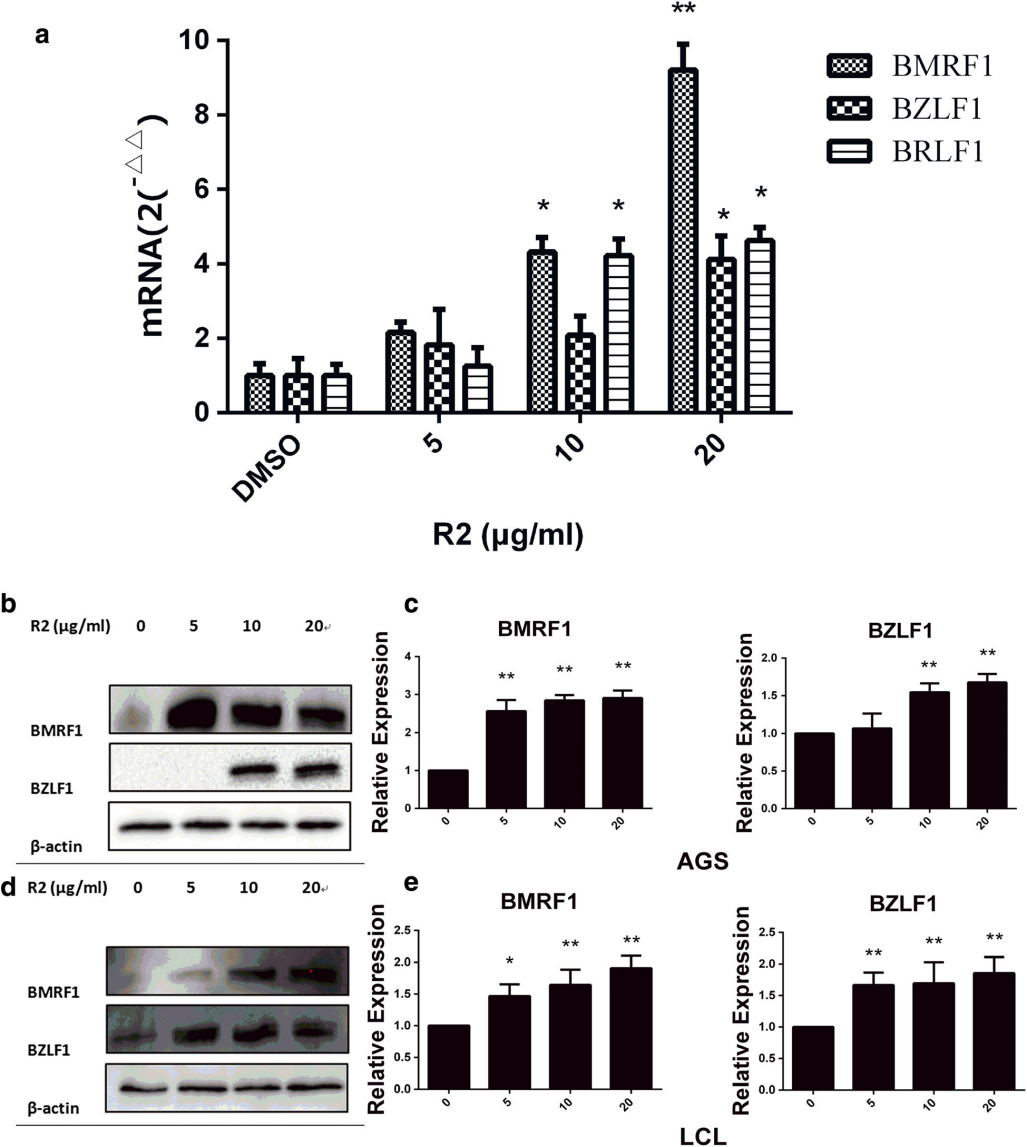
The effects of R2 on the expressions of lytic-related mRNA in AGS-EBV (**a**), the expressions of lytic-related proteins in AGS-EBV (**b** and **c**) and LCL cells (Fig. 5d and Fig. 5**e**) were determined by RT-PCR and western blot. AGS-EBV and LCL cells were treated with R2 (0, 5, 10, 20 μg/mL) for 24 h. The mRNA expressions of BZLF1, BMRF1 and BRLF1 increased after R2 treatment. R2 also increased the protein expressions of BZLF1 and BMRF1. Relative expression levels of EBV lytic-related proteins were showed in Fig. 6c and e. β-actin was used to confirm equal mRNA and protein loading. *P < 0.05 and ** *p* < 0.01 were considered statistically significant

To further study the effects of R2 on EBV lytic induction, we performed RT-PCR to investigate the expression of essential lytic genes after R2 treatment. AGS-EBV cells were exposed to R2 (DMSO, 5, 10 and 20 μg/mL), then the expression levels of immediate early lytic genes (BZLF1 and BRLF1) and early lytic gene (BMRF1) of EBV were tested. As shown in Fig. 5a, the mRNAs expression level of BZLF1, BMRF1 and BRLF1 were increased in AGS-EBV cell line after R2 treatment.

### Effects of R2 on EBV lytic proteins expression in AGS-EBV and LCL cells

We further analyzed the effects of R2 induced the expression of EBV lytic protein BMRF1 and BZLF1 in both AGS-EBV and LCL cells. The two proteins were required for viral polymerase process and essential for lytic EBV replication [11]. As showed, the expression of BMRF1 and BZLF1 in both AGS-EBV (Fig. 5b) and LCL (Fig. 5d) cells increased after R2 treatment. As EBV lytic was a cascade reaction, the expression of BMRF1 and BZLF1 were not exactly the same.

### Evaluation of cell cycle arrest in AGS-EBV cells after R2 treatment

**Fig. 6:**
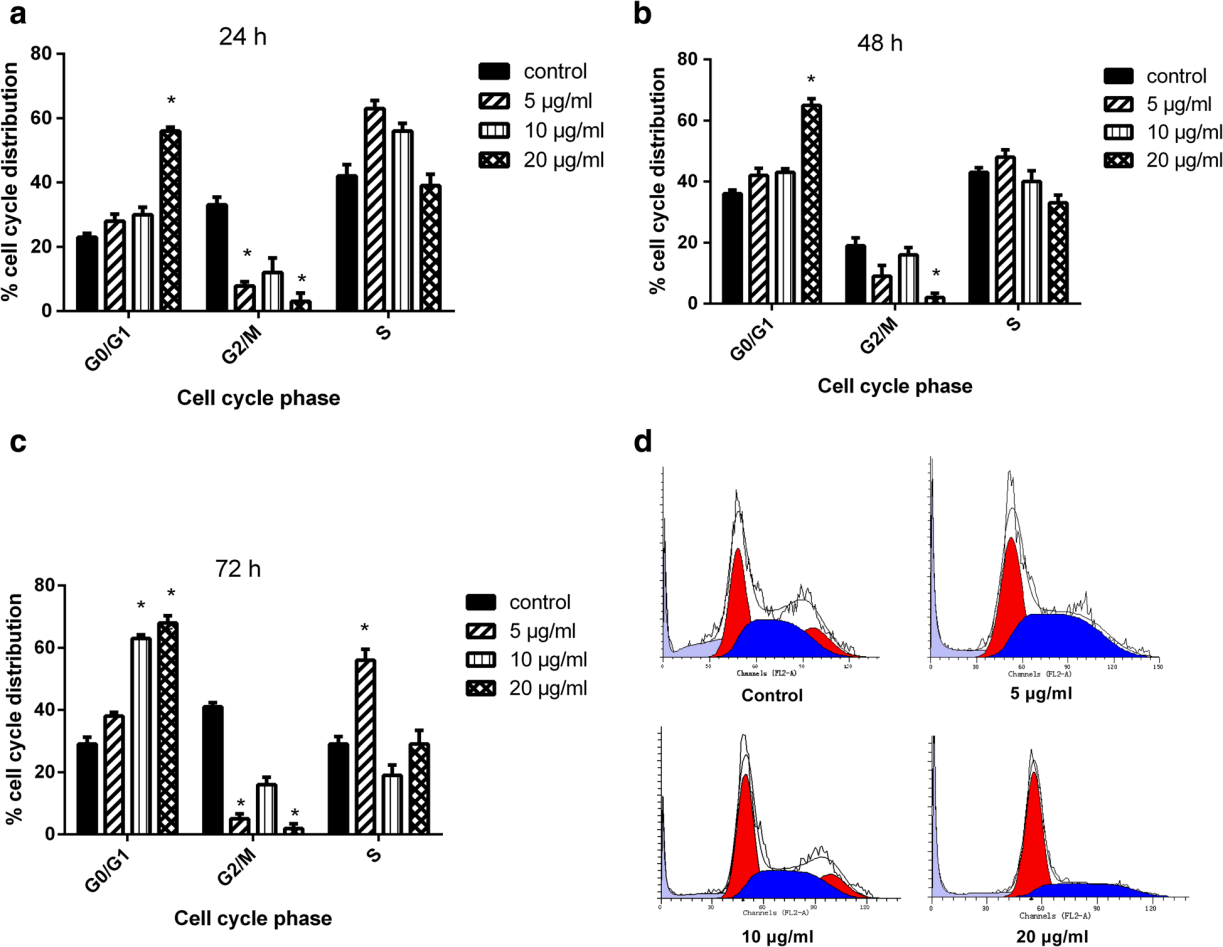
Effects of R2 on cell cycle progression in AGS-EBV cells. Cells were treated by R2 (0, 5, 10, 20 μg/mL) for 24 h, 48 h, 72 h and then analyzed by flow cytometry for cell cycle distribution. Cell cycle distributions after 24 h (**a**), 48 h (**b** and **d**) and 72 h (**c**) treatments in AGS-EBV cells were shown. Results obtained from a representative experiment were shown (*n* = 3). *P < 0.05 when compared with the control group

As R2 showed effects on EBV lytic induction, growth inhibition and apoptosis induction in AGS-EBV cells, we explored its effect on cell cycle arrest. AGS-EBV cells were treated with R2 for 24 h, 48 h and 72 h before flow cytometry analysis. 48 h treatment groups showed G0/G1 phase arrest compared with control groups (Fig. 6b). Similar results were obtained in 24 h treatment group, with a slightly lower number of cells arrested in G0/G1 phase (Fig. 6a). For 72 h treatment group, the number of apoptosis cells increased compared with other 24 h and 48 h groups (Fig. 6c). The distributions of AGS-EBV in cell cycle treated by R2 for 48 h were showed in Fig. 6d. These results suggested the induction of G0/G1 arrest in cell cycle may be a factor of growth inhibition and apoptosis induction in AGS-EBV cells after R2 treatment.

## Discussion

Medicinal plants have played important roles in anti-cancer drug discovery [24]. *Incarvillea compacta* Maxim. is a perennial herb mainly distributed in Tibet and has been for treating stomach disorder for centuries. In the current study, we investigated the anti-cancer effects of *I. compacta* extracts in stomach (AGS, AGS-EBV, BGC-823), LCL, liver (HepG2), leukemia (K562), cervix (HeLa), lung (A549) and prostate (PC3 and DU145) cancer cells. Cytotoxicity studies showed that trichloromethane fraction (R2) possess cell viability inhibition effects in EBV positive cancer cells (AGS-EBV) and wild-type EBV-transformed B-cell lines (LCL) among all the other fractions and other cancer cell lines.

Since R2 showed different inhibition effects on AGS cells and EBV positive AGS cells, we take further investigations on R2. Gastric cancer has been reported to be associated with EBV [31]. EBV persists in EBV positive malignancies as latent form. Entry into the viral lytic cycle is initiated by the expression of two immediate early EBV proteins and one early protein: Zta encoded by BZLF1, and Rta encoded by BRLF1, EAD encoded by BMRF1 [6, 30]. In this study, we explored the ability of R2 inducing EBV lytic cycle in EBV-positive AGS cell lines and critically examined the relationship between viral lytic cycle activation and the induction of apoptosis in AGS-EBV. R2 could potently induce EBV lytic cycle in AGS-EBV and LCL cells. The mRNA expression levels of immediate early (BZLF1 and BRLF1) and early (BMRF1) lytic genes increased in AGS-EBV cells. Western blot and immunofluorescent staining assays showed the BZLF1 and BMRF1 lytic proteins were increased after R2 treatment in both AGS-EBV and LCL cells. These results indicated that R2 was capable of inducing EBV lytic replication in EBV-positive cancer cells.

Our results were consisting with others that the induction of EBV lytic cycle could mediate additional killing of EBV-positive cancer cells [32, 33] We also observed similar phenomenon in AGS-EBV cells after R2 treatment. Hoechst 33342 staining, mitochondrial membrane potential assay and Annexin V-PE/7-AAD assay for apoptosis revealed that R2 could induce apoptosis in AGS-EBV cells. Western blot results showed the expression of Bax increased, while Bcl-2 decreased. Cell cycle arrest is associated with cell death; however which is not the only factor related to cell death. As in our observations, 10 μg/ml of R2 could induce G0/G1 cell cycle arrest, which may be at least partly related to the cell death of AGS-EBV. Other treatments like suberoylanilide hydroxamic acid could induce G2/M arrest and enhanced cell death in EBV positive AGS cells had also been reported [32]. We found that cell cycle related proteins cyclin D1 decreased, while the expression of Rb increased after R2 treatment. Our results also suggested that R2 treatment exhibit less cytotoxic effects on normal cells and other kinds of cancer cells. Further studies of R2 in other EBV positive cancer cell lines such as SNU-719 are required to explore the mechanism and find the molecular target during this process.

## Conclusion

The current research demonstrated that trichloromethane fraction from *Incarvillea compacta* Maxim. roots (R2) inhibit the proliferation of EBV-positive AGS cancer cells by inducing EBV lytic replication, apoptosis and cell cycle G0/G1 arrest, through the regulation of related proteins. Therefore, R2 could be treated as a valuable candidate for further investigation as a possible antitumor agent targeting EBV for gastric cancer therapy.

## Additional file

Additional file 1: Figure S1. UHPLC-MS chromatogram profile of dichloromethane fraction of *I. compacta.* (DOCX 23 kb)

## Abbreviations

DMEM: Dulbecco’s Modified Eagle’s Medium
EBV: Epstein-Barr virus
FBS: Fetal bovine serum
*I. compacta*: *Incarvillea compacta*
IC-BT: *I. compacta* n-BuOH fraction
IC-ET: *I. compacta* ethanolic fraction
IC-R: *I. compacta* H_2_O fraction
IC-TCL R2: Trichloromethane fraction of *Incarvillea compacta* Maxim. Roots
LCL: Lymphoblastoid cell lines
MTT: 3-(4,5-dimethylthiazol-2-yl)-2, 5-diphenyltetrazolium bromide
PI: Propidium iodide
RT-PCR: Real-time polymerase chain reaction
U-HPLC: Ultra-high performance liquid chromatography
