# Supplementary material for: Trichloromethane fraction of Incarvillea compacta induces lytic cytotoxicity and apoptosis in Epstein-Barr virus-positive gastric cancer AGS cells
Source: BMC Complement Altern Med. 2016 Sep 5;16(1):344. doi: 10.1186/s12906-016-1331-6 (PMC5011811; doi:10.1186/s12906-016-1331-6)
Supplement: Additional file 1: Figure S1. — UHPLC-MS chromatogram profile of dichloromethane fraction of I. compacta. (DOCX 23 kb) [file 12906_2016_1331_MOESM1_ESM.docx]

**Supplementary information**

**Supplementary Figure 1** UHPLC-MS chromatogram profile of dichloromethane fraction of *I. compacta.*
